# The Association of Fetal and Early Childhood Growth with Adult Mental Distress: Evidence from the Johns Hopkins Collaborative Perinatal Study Birth Cohort

**DOI:** 10.3389/fpsyt.2013.00096

**Published:** 2013-09-05

**Authors:** Aaron A. Alford

**Affiliations:** ^1^Battelle Center for Analytics and Public Health, Arlington, VA, USA

**Keywords:** depression, developmental origins of health and disease, fetal growth retardation, mental distress, early growth

## Abstract

**Objectives:** Early childhood physical growth may have an impact on the development of adult mental distress. The primary objectives were to (1) assess the association of early growth in weight (adjusted for height) with adult mental distress, and (2) determine if specific sub-types, or patterns, of early physical growth are associated with adult mental distress.

**Methods:** Subjects were all Johns Hopkins Collaborative Perinatal Study cohort subjects with complete birth size information that successfully completed the Pathways to Adulthood follow-up in early adulthood. Variability in the timing of growth in weight adjusted for height from birth to age 7.5 years was taken into account using a non-hierarchical linear model. Two critical periods of growth were considered as tertiles of change in weight adjusted for height from birth to age 7 and birth to age 1 year. Mental distress in adulthood (ages 29–32) was measured using the General Health Questionnaire (GHQ-28).

**Results:** Small for gestational age subjects were at increased risk of later mental distress, but not uniformly so. Those born with low weight and length for gestational age were a distinct subgroup of those born small for gestational age, and had unique patterns of risk for adult mental distress when early growth was considered.

**Conclusion:** Acceleration and deceleration in weight for height change is associated with mental distress over multiple periods of early life and acts differentially between those periods. Furthermore, the association of early childhood growth with the likelihood of adult mental distress is dependent on prenatal growth.

## Introduction

Fetal and childhood development has lasting effects on adolescent and adult health. Nearly all of the biological processes and structures that we rely on in adulthood are set in motion or created during early life. Deviations from normal development can result in processes or structures that are immediately recognizable as pathological, such as gross physical abnormalities. However, some deviations from normal development instigate processes that do not result in recognizable pathologies until much later in life.

Under the Thrifty Phenotype Hypothesis, a fetus faced with the challenge of poor nutrition or oxygen, or incapacity to utilize nutrition, becomes increasingly metabolically efficient, or “thrifty” (1). After the challenge has passed, the “thrifty” adaptation persists, resulting in increased risk of Type II diabetes, metabolic syndrome, and coronary artery disease in later life, regardless of adult risk factors.

Fetal growth restriction (FGR) is the key component of the Thrifty Phenotype Hypothesis (1). FGR is a physical deviation from the genetically programed growth potential of the fetus under ideal conditions, but various definitions exist for defining FGR within populations (2, 3). FGR may take place during any phase of fetal development, with the effects on the fetus varying substantially with the timing, duration, and cause of the restriction (2). Programing occurs when a system or organ does not have the capacity to fully return to its prior state or level of function after it has been disrupted during a critical period of development (4).

Anthropometric manifestations of FGR at birth are heterogeneous, but have traditionally been thought to indicate three broad groups of growth restriction: length restriction, weight restriction, and symmetric restriction (5–8). Length restriction results in infants with a reduced length for gestational age, with appropriate weight for gestational age (5). Length growth restriction most likely occurs early in gestation when the fetus experiences the greatest longitudinal skeletal growth velocity (8, 9). Weight restriction results in infants with a reduced weight for gestational age, with appropriate length for gestational age. Infants experiencing weight restriction are thought to have experienced restriction in the final weeks prior to term birth when weight growth is at its greatest (8, 9). Weight and length restriction that occur alone are generally classified as asymmetric growth restriction. This general rubric is formalized as the “timing hypothesis” (8, 9).

The nomenclature of symmetric and asymmetric restriction is rooted in the hypothesized differential effect of growth restriction on organ development (10, 11). Human ultrasonography (11, 12), pathology (13), and experimental animal studies (14) indicate that the fetus can selectively reroute blood flow from less immediately vital organs to the brain when challenged with hypoxia or under nutrition or other forms of stress. Asymmetry results when the brain is “spared,” but other systems fail to grow to gestationally appropriate size. When brain sparing fails, or the insult occurs in the first trimester both the head and the remainder of the body are thought to be symmetrically reduced in size.

Postnatal growth may modify, or at the very least indicate, the course of fetal programing. Like fetal growth, postnatal, and early childhood physical growth is highly ordered at the population level, with its own series of associated critical periods that have proven to be liable to programing (15, 16). Throughout early childhood, weight and height growth for the individual has a strong tendency to remain in the same percentile (canalization) relative to peers of the same biological age (16). Canalization is widely held as evidence of genetic control of the growth process, but does not apply as strongly to the first 18 months of life. During this period, the infant is free from the constraints of the womb and will seek to realign with its genetic potential. Realignment through accelerated growth, or catch-up growth, has been found to occur in over 30% of infants in a well-nourished market society (17). Nearly 30% experience decelerated, or catch-down, growth. Approximately 60% of infants born small for gestational age experience catch-up growth (18). After 2 years of age, significant acceleration or deceleration in growth is generally held to be the result of pathology.

Increasing evidence indicates that the adaptive fetal response to reduced nutrition, or programing, includes an alteration of functioning in the Hypothalamic-Pituitary-Adrenal axis (HPA-axis), with substantial alterations in cortisol regulation (3, 19). In turn, HPA-axis function is known to play a key role in the etiology of hypertension, metabolic syndrome, stress response, and affective disorders (19, 20).

Two large longitudinal European studies provide some of the most compelling evidence that FGR and later growth may interact to impact later mental health. Unfortunately, the two studies provide competing conclusions as to the direction of effect. Analysis of data from a 1918 British cohort found decreasing risk of depression with increasing birth weight across four birth weight categories among men (adjusted for weight at 1 year, as well as predictors of depression among the elderly) (21). Weight at 1 year was not significant when adjusting for birth weight. This study also tested the association between method of feeding as an infant and depression at 63 years. An analysis of the 1958 National Child Development Study cohort found that birth weight *z*-scores, adjusted for gestational age, were significantly inversely associated with psychological distress at ages 23 and 42, but not at age 33. Standardized weight gain from birth to age 7 was also significantly inversely related to adult mental distress. When weight gain was adjusted for, the one unit relationship between increased birthweight and decreased symptoms of distress increased, suggesting that faster growth has a protective effect, or slower growth has a deleterious effect (22).

Multiple periods of growth were included in the present study as part of a broader hypothesis that growth over different periods cannot be treated as a single uniform construct. To consider only one period of growth may hide or ignore the effects of other periods of growth. For example, significant relationships between mental health outcomes and early growth through age 1 year would be obscured if only including a measure of growth to age 8 years. Considering a single period of early growth also ignores the fact that the success or failure of growth during one period can affect the timing and success of later growth. To test the hypotheses of the specificity of the effects of each period of growth the current study included four potential trajectories of intrauterine growth and physical growth over two significant periods of early childhood. By including multiple periods of growth, it may be possible to more precisely elucidate patterns of early physical growth that increase or attenuate risk for adult mental distress.

The present paper addresses the following research questions:

- Are trajectories of weight for height growth related to adult mental distress?
- Is the relationship between weight for height and mental distress different when adjusting for different periods of growth from birth to age 7?
- What combinations of weight for height growth and birth size best predict adult mental distress?

## Materials and Methods

### Subjects

The current study focuses on 1680 adults (Table 1) followed during the Johns Hopkins Collaborative Perinatal Study (JHCPS) and a subsequent follow-up study, the Pathways to Adulthood Study (PAS). The current study sample is comprised of all JHCPS children for whom stratified estimates of low weight and short height for gestational age were available and who were successfully followed during the Pathways follow-up. Of the 4025 pregnancies enrolled in the JHCPS, 1758 were successfully followed during the Pathways follow-up. In all, 1680 subjects received the full Pathways interview and met the inclusion criteria for the stratification procedure described above. These subjects are the primary focus of the current study. From the PAS sampling frame of 2,694 subjects, the current study has achieved a retention rate of 62%. Of those who successfully received the full Pathways interview, the current sample has retained over 95%.

**Table 1:** **Frequency distributions of predictors and outcomes by sex; bivariate relationship of mental distress and study variables**.

	Female, *N* (%)	Male, *N* (%)	Mental distress: odds ratio (U95% CI | L95% CI)
Study sample (*n* = 1680)	918	762	
**G-2 ADULT MENTAL DISTRESS**			
Present	268 (65.53)	141 (34.47)	–
Absent	650 (51.14)	621 (48.86)	–
**G-2 BIRTH CHARACTERISTICS**			
Weight for gestational age
Low weight	127 (54.51)	106 (45.49)	**1.40 (1.03 | 1.91)**
Normal weight	791 (54.66)	656 (45.34)	Ref
Length for gestational age
Short length	163 (53.09)	144 (46.91)	0.80 (0.60 | 1.08)
Normal length	755 (54.99)	618 (45.01)	Ref
Length for gestational age (9% threshold)
Short length	143 (51.81)	133 (48.19)	0.88 (0.65 | 1.20)
Normal length	775 (55.20)	629 (44.80)	Ref
Combinations of weight and length for gestational age
Appropriate weight and length	708 (54.88)	582 (45.12)	Ref
W-SGA	47 (56.63)	36 (43.37)	**1.66 (1.04 | 2.66)**
L-SGA	83 (52.87)	74 (47.13)	**0.56 (0.35 | 0.88)**
W&L-SGA	80 (53.33)	70 (46.67)	1.16 (0.80 | 1.70)
Combinations of weight and length for gestational age (9% threshold)
Appropriate weight and length	727 (55.12)	593 (44.88)	Ref
W-SGA	48 (56.47)	37 (43.53)	**1.72 (1.08 | 2.74)**
L-SGA	64 (50.00)	64 (50.00)	0.66 (0.41 | 1.06)
W&L-SGA	79 (53.38)	69 (46.62)	1.17 (0.80 | 1.72)
Low birth weight (≤2500 g)
Normal	779 (54.25)	657 (45.75)	Ref
Low	139 (56.97)	105 (43.03)	1.04 (0.76 | 1.43)
Preterm birth (<37 weeks)
Normal	774 (55.25)	627 (44.75)	Ref
Preterm	144 (51.61)	135 (48.39)	1.17 (0.88 | 1.57)
Race
White	752 (54.65)	624 (45.35)	Ref
Black	166 (54.61)	138 (45.39)	**0.66 (0.50 | 0.87)**
Sex
Female	918	–	Ref
Male	–	762	**0.55 (0.44 | 0.70)**
**G-2 GROWTH**			
Tertiles of weight for height gain, from birth to age 7.5 years
Lower tertile	320 (57.25)	239 (42.75)	0.93 (0.70 | 1.22)
Middle tertile	295 (52.68)	265 (47.32)	Ref
Highest tertile	303 (54.01)	258 (45.99)	1.09 (0.83 | 1.42)
Tertiles of weight for height gain, from birth to age 1 year
Lower tertile	346 (61.90)	213 (38.10)	0.93 (0.71 | 1.22)
Middle tertile	313 (55.89)	247 (44.11)	Ref
Highest tertile	259 (46.17)	302 (53.83)	0.77 (0.58 | 1.01)
Mean gain in kg, Mean(SD)	6.41 (0.36)	6.49 (0.38)	0.81 (0.59 | 1.09)
**MATERNAL CHARACTERISTICS**			
Pre-pregnancy height
<1.55 m	177 (57.28)	132 (42.72)	Ref
1.55–1.61 m	287 (54.77)	237 (45.23)	0.89 (0.65 | 1.21)
1.62–1.66 m	248 (56.24)	193 (43.76)	0.75 (0.54 1.04)
>166 m	196 (50.52)	192 (49.48)	**0.68 (0.48 | 0.97)**
Missing	10 (55.56)	8 (44.44)	
Pre-pregnancy BMI
Low (<19.8 kg/m^2^)	207 (53.49)	180 (46.51)	0.92 (0.70 | 1.22)
Normal (19.8–26 kg/m^2^)	481 (53.80)	413 (46.20)	Ref
High (26.1–29 kg/m^2^)	99 (58.93)	69 (41.07)	0.83 (0.56 | 1.22)
Obese (>29 kg/m^2^)	108 (56.25)	84 (43.75)	0.74 (0.51 | 1.09)
Missing	23 (58.97)	16 (41.03)	
Parity
Nulliparous	250 (53.53)	217 (46.47)	Ref
Primaparous	146 (52.52)	132 (47.48)	0.99 (0.71 | 1.39)
Multiparous	315 (58.33)	225 (41.67)	0.86 (0.65 | 1.15)
Grand multiparous	207 (52.41)	188 (47.59)	0.90 (0.66 | 1.23)
Mother’s education
≤8th Grade	273 (58.84)	191 (41.16)	Ref
Some high school	379 (53.01)	336 (46.99)	0.96 (0.73 | 1.25)
High school	188 (52.81)	168 (47.19)	**0.65 (0.46 | 0.90)**
Greater than HS	58 (50.43)	57 (49.57)	0.76 (0.47 | 1.24)
Unknown	20 (66.67)	10 (33.33)	
Maternal smoking
Non-smoker	545 (54.34)	458 (45.66)	Ref
<4 per day	132 (52.80)	118 (47.20)	1.13 (0.82 | 1.55)
5–14 per day	137 (55.47)	110 (44.53)	1.12 (0.82 | 1.55)
15 or more per day	101 (57.39)	75 (42.61)	0.95 (0.65 | 1.38)
Missing	3 (75.00)	1 (25.00)	
Maternal age
<25 years	538 (54.45)	450 (45.55)	Ref
25–34 years	292 (55.73)	232 (44.27)	0.93 (0.72 | 1.18)
35 years and above	88 (52.38)	80 (47.62)	0.84 (0.57 | 1.25)
**G-2 ADULT CHARACTERISTICS**			
G-2 Adult BMI
<25	427 (54.19)	361 (45.81)	Ref
25–29.9	238 (47.41)	264 (52.59)	0.87 (0.67 | 1.14)
≥30	216 (63.72)	123 (36.28)	1.20 (0.90 | 1.61)
Missing	37 (72.55)	14 (27.45	
G-2 household or individual income
$0–$4,999	145 (57.31)	108 (42.69)	Ref
$5,000–$14,999	218 (57.22)	163 (42.78)	0.75 (0.54 | 1.05)
$15,000 or more	555 (53.06)	491 (46.94)	**0.41 (0.30 | 0.55)**
G-2 smoking status
Never smoked	278 (54.30)	234 (45.70)	Ref
Ever smoked	640 (54.79)	528 (45.21)	**1.67 (1.29 | 2.17)**
Maintained overweight
No	844 (54.03)	718 (45.97)	Ref
Yes	59 (59.60)	40 (40.40)	**1.79 (1.16 | 2.75)**
Missing	15 (78.95)	4 (21.05)	
Illicit drug use in the past 30 days
No	890 (55.59)	711(44.41)	Ref
Yes	28 (35.44)	51 (64.56)	**1.76 (1.09 | 2.83)**

Unadjusted odds ratios. Bolded OR’s significant at α = 0.05.

### Outcome and growth measures

The primary outcome measure is mental distress as recorded by the GHQ-28. The GHQ-28 is a self-administered questionnaire commonly included in population surveys to identify persons with any psychiatric disorder (23, 24). A positive indication of mental distress on the GHQ-28 is highly correlated with a clinical rating of depression (*r* = 0.73 when the information from all four scales is combined) (23).

**Figure 1:**
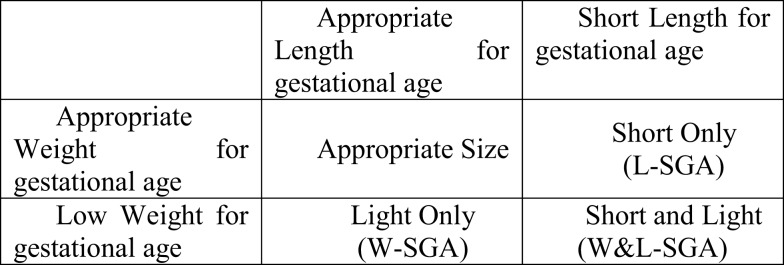
**Mutually exclusive combinations of short length and low weight for gestational age**.

For this study, low weight for gestational age was defined as those at or below the lowest 10% of birth weights (within strata defined by week of gestational age, race, sex, and parity). Likewise, short length for gestational age was defined as those at or below the lowest 10% of birth lengths (within strata defined by week of gestational age, race, sex, and parity). Stratified birth weight and length were included as dichotomous measures to identify subjects most likely to have experienced some form of growth disruption prior to birth (25). Theoretically, combining these two measures result in the capacity to identify significant *in utero* growth disruption of the three distinct forms shown in Figure 1. Those who are short for gestational age alone or light for gestational age alone, represent two different forms of asymmetrical growth restriction. Those small for gestational age on *both* measures experienced symmetric growth restriction.

The full heterogeneity of birth size was captured by a categorical variable with the four mutually exclusive combinations of the indicators light weight for gestational age and short length for gestational age represented in Figure 1. For the remainder, the abbreviations noted in Figure 1 will be used to identify persons in each of the three growth restricted conditions. The presence of *only* low weight for gestational age is indicated by W-SGA. The presence of both simultaneously is indicated by W&L-SGA. The terms low weight for gestational age and short length for gestational age are used in a more traditional sense. “Low weight for gestational age” denotes subjects with the lowest 10% of all birthweights (within strata defined by gestational age, race, sex, and parity), regardless of birth length. “Short length for gestational age” denotes subjects with the lowest 10% of all birth lengths (within strata defined by gestational age, race, sex, and parity), regardless of birth weight.

To estimate change in weight for height from birth to age 7.5 years we used a two step process. First, for each individual we estimated weight, adjusted for height, using a multiple linear regression of weight on height. This process was repeated for each of the four periods of measurement from birth to 7.5 years. In a separate analysis, using the adjusted weight scores as the dependent variable, we examined trajectories of weight for height growth over time. From the best fitting growth analysis we derived categorical variables of change in weight for height for the periods spanning birth to age 1 and birth to age 7.5. This process allowed for the imputation of missing growth information and accounted for the variability of timing of measure for anthropometric measures.

### Analysis frame

Multiple logistic regression models were used to test whether adult mental distress is related to birth size, growth from birth to ages 1 or 7, or an interaction of the factors. An initial comparison model of explanatory variables was selected based on the unadjusted odds ratios presented in Table 1, and the current literature. In order to test the relative contributions of weight, length, weight for height growth, and their interactions, each term or combination of terms were added to the base model. The relative contributions of the added terms were then tested by comparison to the base model.

## Results

In the most appropriate logistic regression model (Table 2), slow weight for height gains to age 7.5 years decreased the likelihood of adult mental distress among term infants without the combination of short length and low weight for gestational age (W&L-SGA) (OR = 0.71, 95% CI = 0.50 | 0.995), all else held constant. Among the other lower order growth terms associated with an interaction term, none were significantly associated with adult mental distress.

**Table 2:** **Final multiple logistic regression model of the relationship between early growth and later mental distress**.

	Final model (odds ratios and 95% CI’s)
G-2 characteristics at birth
W-SGA
Normal weight (with normal growth to1 year)	Ref
Low weight (with normal growth to1 year)	**3.30 (1.61 | 6.78)**
L-SGA
Normal length	Ref
Short length	**0.44 (0.26 | 0.73)**
W&L-SGA with normal growth to 7.5 years
Normal	Ref
Low weight and short length	0.57 (0.19 | 1.70)
Preterm birth (<37 weeks)
Normal	Ref
Preterm	0.95 (0.52 | 1.73)
Sex
Female	Ref
Male	**0.43 (0.28 | 0.67)**
Race
White	Ref
Black	**0.64 (0.46 | 0.88)**
**G-2 GROWTH**	
Tertiles of weight for height gain, from birth to age 7
Lower tertile	**0.71 (0.50 | 0.995)**
Middle tertile	Ref
Highest tertile	0.83 (0.59 | 1.17)
Tertiles of weight for height gain, from birth to age 1 year
Lower tertile	1.02 (0.70 | 1.50)
Middle tertile	Ref
Highest tertile	0.71 (0.47 | 1.07)
Interactions with growth to age 1 year
W-SGA × lower tertile	0.48 (0.22 | 1.05)
W-SGA × middle tertile	Ref
W-SGA × highest tertile	**0.27 (0.10 | 0.74)**
Sex × lower tertile	0.93 (0.50 | 1.75)
Sex × middle tertile	Ref
Sex × highest tertile	**2.04 (1.11 | 3.76)**
Interactions with growth to age 7.5 years
Low weight and short length × lower tertile	**3.18 (1.04 | 9.65)**
Low weight and short length × middle tertile	Ref
Low weight and short length × highest tertile	**3.97 (1.31 | 12.05)**
Preterm × lower tertile	2.15 (0.97 | 4.79)
Preterm birth × middle tertile	Ref
Preterm birth × highest tertile	0.69 (0.30 | 1.59)
Maternal characteristics
Pre-pregnancy height
<1.55 m	Ref
1.55–1.61 m	0.98 (0.70 | 1.39)
1.62–1.66 m	0.78 (0.54 | 1.13)
>166 m	0.76 (0.52 | 1.12)
Mother’s education
≤8th Grade	Ref
Some high school	1.14 (0.85 | 1.53)
High school	0.77 (0.53 | 1.12)
Greater than HS	1.09 (0.63 | 1.86)
Parity
Nulliparous	Ref
Primaparous	1.00 (0.69 | 1.45)
Multiparous	0.81 (0.59 | 1.11)
Grand multiparous	0.92 (0.65 | 1.29)
Maternal smoking
Non-smoker	Ref
Less than 4 per day	1.21 (0.86 | 1.71)
5–14 per day	0.87 (0.61 | 1.24)
15 or more per day	0.76 (0.49 | 1.18)
G-2 Adult characteristics
Maintained overweight
No	Ref
Yes	**1.91 (1.16 | 3.15)**
G-2 household or individual income
$0–$4,999	Ref
$5,000–$14,999	0.71 (0.49 | 1.02)
$15,000 or more	**0.43 (0.31 | 0.60)**
G-2 smoking status
Never smoked	Ref
Ever smoked	**1.41 (1.06 | 1.87)**
Illicit drug use in the past 30 days
No	Ref
Yes	**1.84 (1.09 | 3.12)**
	*N* = 1612

Bolded entries represent statistically significant findings.

Weight for height growth was a significant predictor of mental distress when considered among groups with a priming condition at birth, such as low weight for gestational age. Subjects with W-SGA and normal growth to age 1 year were three times more likely to experience mental distress in adulthood, compared to all subjects with normal growth to age 1 year (OR = 3.30, 95% CI = 1.61 | 6.78), after controlling for early life and adult covariates. Subjects with W-SGA and strong growth to age 1 were less likely to experience adult mental distress, compared to W-SGA with normal growth (ROR = 0.27, 95% CI = 0.10 | 0.74) after controlling for early life and adult covariates.

Those born with low weight and length for gestational age (W&L-SGA) are a distinct subgroup of those born small for gestational age, and have unique patterns of risk for adult mental distress when early growth is considered. W&L-SGA subjects with normal growth to age 7.5 years were no more likely to experience adult mental distress than non-W&L-SGA subjects with normal growth. However, after controlling for birth and adult covariates, W&L-SGA subjects were more likely to have mental distress if they experienced any subsequent deviation in weight for height gains to age 7.

Males with accelerated growth to age 1 were twice as likely to experience mental distress as males with normal growth to age 1 (ROR = 2.04, 95% CI = 1.11 | 3.76), all else held constant. Males with normal weight for height growth to age 1 were less than half (OR = 0.43, 95% CI = 0.28 | 0.67) as likely to experience mental distress relative to females with normal growth age 1, after controlling for all other covariates.

Blacks were half (OR = 0.64, 95% = 0.64 | 0.88) as likely as whites experience mental distress, all else equal. The remainder of maternal and birth characteristics had no association with adult mental distress. Some evidence presented above suggested that weight for height growth to age 7 years altered the likelihood of mental distress among those born preterm. However, after the inclusion of adult covariates, no evidence of this effect remained in the final model.

In accordance with the bivariate associations reported above, all of the adult covariates maintained a significant relationship with adult mental distress. Most surprisingly, maintained obesity maintained a strong association with adult mental distress, independent of the other adult covariates. Subjects with maintained overweight (overweight at ages 7.5 years and early 30s) were 91% (OR = 1.96, 95% CI = 1.16 | 3.15) more likely to have mental distress in adulthood compared to all other subjects, after controlling for birth and adult covariates.

Table 3 is a visual reinterpretation of the main findings from Table 2. Table 2 reports several interactions, which are notoriously difficult to interpret. The visual map in Table 3 parses out the core findings from the interactions and main effects in the logistic regression model reported in Table 2. Direct effects of birth size alone are listed in the first column. The remaining columns show the interactions of birth size and later physical growth. Bolded text represents statistically significant effects.

**Table 3:** **The combinations of growth restriction and early growth considered, along with their observed effect on mental distress in the Pathways to Adulthood Study subsample of the JHCPS cohort**.

		Direct effect of birth size alone	Weight for height growth to 1 year		Weight for height growth to 7.5 years	
			Low growth	High growth	Low growth	High growth
Birth size for gestational age	*Low* weight, normal length	**Increased mental distress**	No effect	**Decreased mental distress**	No effect	No effect
	normal weight, *short* length	No effect	No effect	No effect	No effect	No effect
	*Low* weight, *short* length	No effect	No effect	No effect	**Increased mental distress**	**Increased mental distress**

Bolded entries represent statistically significant findings.

## Discussion

In the present study, low weight gain prior to birth is a priming event for adult mental distress. Even after controlling for the effects of gestational age, early life covariates, and adult risk factors, weight for height gain in early childhood acts to mitigate or elevate the likelihood of mental distress only among those born with low weight for gestational age. The effect of early childhood growth depends on whether the subject was born asymmetrically small on weight alone (W-SGA) or symmetrically small on both weight and length (W&L-SGA). Furthermore, the period of growth associated with altered likelihood of mental distress in these groups varies.

We hypothesized that early and fast weight for height growth among W-SGA, L-SGA, and W&L-SGA would be a protective factor, as long as they did not continue to accelerate past age 1 year. Strong weight for height gain to age 1 year was protective for mental distress among W-SGA, other factors held constant, as can be seen in the second column of Table 3 above. Pediatrics has long held that catch-up growth, or realignment to genetic potential, occurs within the first 18 months of life among those born small for gestational age (26). While the role of catch-up growth as a risk/protective factor for adult disease has been hotly contested, a growing body of evidence suggests that successful early catch-up growth is beneficial among small for gestational age infants (27).

The observed protective effect of early accelerated growth found in this study is at odds with the only other cohort study to consider growth to age 1 year and later mental distress. In the 1920s British cohort, male subjects with the lowest birthweights and highest weight at 1 year of age were found to be at increased risk of depression at age 68 years (21). They also found that male subjects with the highest birthweights and the lowest weights at age 1 year were at increased risk of mental distress. Unfortunately, the numeric values of these findings are not reported. Their findings suggest that low growth to age 1 year is a protective factor for depression and high growth is a risk factor for depression, depending on your condition at birth. However, their modeling approach did not take gestational age or weight change to age 1 into account.

These exclusions may have biased their findings in several ways. Gross weight at any age does not quantify growth, when compared to a prior period with varying weight. This is especially true over the period from birth to 1 year. Though growth is rapid during this period, the short duration makes differences in weight relatively small. The effect is compounded by a lack of adjustment for biological age, a standard procedure when comparing the growth of individuals in early childhood. The cohort considered was born in the 1920s suggesting that too few preterm infants survived to consider including a measure of gestational age. However, this also indicates that the inference reached must be considered with care. If one assumes that only fundamentally healthy infants could survive the perinatal period, due to a low standard of care, then we would not necessarily expect a high degree of comparability to a cohort receiving modern obstetric care.

We hypothesized that small for gestational age subjects who grew fast after age one would experience an increased risk of mental distress. This expectation was driven by findings concerning metabolic syndrome, wherein the greatest increase in risk has been seen among those born small, grow slowly, and who are larger than their peers in late childhood (27). Our findings indicate that any strong deviation in weight for height to age 7.5 years increases likelihood of mental distress only among the W&L-SGA. We did not predict an increased likelihood of mental distress among W&L-SGA with poor weight for height growth to age 7.5 years.

It is generally held that the physical growth of symmetrically small infants (W&L-SGA) mirrors that of subjects born appropriate size for gestational age, in the first 18–30 months of life (25). However, recent work in the field has begun to recognize that growth deviations beyond 18 months may alter the risk conferred by interruptions in prenatal growth (27). More importantly, the alterations in risk may differ between early catch-up/catch-down growth (traditionally defined catch-up growth) and later growth deviations (27). Under this expanded view, there is little systematic evidence concerning patterns of long-term catch-up growth among symmetrically growth retarded infants past 30 months, due to the limited period of observation included in prior studies. In the present case, fast growth to age 7.5 years may reflect the result of early programed metabolic changes leading to metabolic syndrome. A recent article comparing the pathophysiologic effects of depression and metabolic syndrome found that both seem to give rise to similar cardiovascular pathology, and operate through many of the same mechanisms (28).

The unexpected finding may be due to the unique growth definition used in the current study. Weight for height, the primary growth measure in the current study, is a common measure of body composition. More precisely, weight for height is an index of adiposity. Adiposity reaches its lowest point around the fifth year of life and then begins to rebound thereafter. Small for gestational age status has been associated with perturbations in the timing of adipose rebound among small for gestational age children (29).

Unfortunately, no studies have measured this effect among symmetrically small for gestational age infants alone. The increased risk among W&L-SGA with poor weight for height growth to 7.5 years may be associated with later adipose rebound in this group. By extension, this group may also experience weight for height catch-up after the period considered. Under this scenario, they would show a smaller net increase in weight for height than their peers to 7.5 years because their rebound was late. In other words, they are the thinnest at age 7.5 years because they have failed to rebound years after their peers began to fatten. As stated previously, the pattern of slowed early development and late catch-up growth has generally been associated with poorer health outcomes in the Developmental Origins literature (27). By extension, this may indicate that the biological model of increased cortisol secretion associated with failed catch-up among small for gestational age infants, presented above, may also underlie the increased likelihood of mental distress among the slowest growing W&L-SGA subjects.

The present analysis was designed, in part, to distinguish between the effects of symmetrically and asymmetrically small for gestational age infants. Growth trajectories, though interesting in their own right, also provided a second source of information concerning those born small for gestational age. We originally hypothesized that symmetrically small infants would display even greater risk of mental disorder than asymmetrically small infants. Traditionally, symmetrically small infants were thought to be fundamentally compromised (11).

In our sample, symmetrically small infants comprised 65% of those born small for gestational age. They also exhibited no increased risk for mental distress prior to considering later growth. If W&L-SGA infants were intrinsically compromised, we would expect a higher rate of mental distress regardless of growth. We did not observe this pattern among the W&L-SGA. However, we did observe precisely that pattern among W-SGA, or asymmetrically growth restricted infants. Regardless of growth, W-SGA subjects displayed increased odds of mental distress.

The increased risk among those with low weight for gestational age (W-SGA) alone remained when controlling for the other three intrauterine growth trajectories specified by the timing hypothesis (8). In other words, the asymmetrically small infants, alone, were responsible for the increased odds of mental distress among those born small for gestational age. The observed pattern is the reverse of that expected by the older understanding, but matches well with the newer understanding of asymmetric growth restriction as an indication of fundamental compromise.

Findings from the current study shed some light on the utility of using birth length for gestational age as a predictor of mental distress. When considered only in the presence of early life covariates and weight for height growth, length did not predict mental distress. Only when combined with birthweight for gestational age did length show a significant protective effect. According to the timing hypothesis, short length at birth would indicate growth restriction during the second trimester, the hypothesized period of greatest length growth velocity. By extension, our findings would then indicate that a transient, second trimester, growth restricting event reduces the likelihood of adult mental distress, all else held equal. However, this proposition is unlikely and the direction of association does not agree with prior findings concerning suicidal behavior (30).

### Limitations

The current study applied several novel methods to create a developmentally appropriate model of early growth. Growth was operationalized as weight for height change rather than gross or relative weight at a given point in childhood. This approach explicitly addresses the fact that birthweights vary. In other words, not everyone begins life at the same weight. Using weight for height, rather than gross weight gain, allowed us to control for one of the three primary components of physical anthropometry (weight, height, composition). Doing so also allowed for some inference concerning changing composition. Variance in timing of physical measurement was accounted for using a linear growth model, which also allowed for the estimation of a small number of missing growth measurements. The current study is also the first to distinguish between symmetrically and asymmetrically small for gestational age infants and estimate their likelihood for exhibiting adult mental distress.

Data from gold standard measures of intrauterine growth restriction were not available in the current study and thus we have relied on measures of birth length and weight for gestational age. Clinically, small for gestational age status and FGR are different entities, but the increase in risk for many antenatal outcomes seen among SGA infants is most likely due to the high proportion of growth restricted infants among SGA births (Royal College of Obstetricians and Gynaecologists, 2002). Therefore, we cannot make the claim that FGR is directly related to an increase in risk for mental distress. However, the findings concerning birth size for gestational age, and later growth, are consistent with the current understanding of the effects of constrained fetal growth and altered antenatal growth on later health outcomes.

## Conclusion

The primary scientific contribution lies in the finding that acceleration and deceleration in weight for height change acts on mental distress over multiple periods of early life and acts differentially between those periods. The impact of early childhood growth on the likelihood of adult mental distress is dependent on prenatal growth. Findings from the present study suggest that low weight gain prior to birth is a priming event for adult mental distress. Even after controlling for the effects of gestational age, early life covariates, and adult risk factors, weight for height gain in early childhood acts to mitigate or elevate the likelihood of mental distress only among those born with low weight for gestational age. The effect of early childhood growth depends on whether the subject was born asymmetrically small on weight alone (W-SGA) or symmetrically small on both weight and length (W&L-SGA). Furthermore, the period of growth associated with altered likelihood of mental distress in these groups varies.

Future studies of the Developmental Origins Hypotheses and mental disorder are warranted to elucidate environmental or physical agents that may act through changes in weight for height deceleration/acceleration. The introduction of gold standard measures of FGR and adequately dense follow-up of childhood growth are particularly needed in future cohort studies. Better identification and measurement of both growth restriction and postnatal growth patterns will allow for the disentanglement of intrauterine versus postnatal causes of growth disruption. While current theory suggests that changes in the HPA-axis may underlie this process, early feeding practices, socioeconomic status, or other environmental changes are likely to contribute equally. By investigating these external influences, and how they interact with growth and later mental disorder, it may be possible to identify postnatal pathways for intervention.

## Conflict of Interest Statement

The author declares that the research was conducted in the absence of any commercial or financial relationships that could be construed as a potential conflict of interest.
